# Identification of neutrophil extracellular traps and crosstalk genes linking inflammatory bowel disease and osteoporosis by integrated bioinformatics analysis and machine learning

**DOI:** 10.1038/s41598-023-50488-4

**Published:** 2023-12-27

**Authors:** Gang Xu, Wanhao Zhang, Jun Yang, Na Sun, Xiaochen Qu

**Affiliations:** 1 Department of Orthopaedics, First Affiliated Hospital of Dalian Medical University, Dalian, Liaoning Province China; 2 Key Laboratory of Molecular Mechanism for Repair and Remodeling of Orthopaedic Diseases, Dalian, Liaoning Province China; 3 Department of Pharmacy, The Third People’s Hospital of Dalian, Dalian, Liaoning Province China

**Keywords:** Computational biology and bioinformatics, Genetics, Immunology, Metabolic disorders, Diagnostic markers, Predictive markers, Prognostic markers

## Abstract

Musculoskeletal deficits are among the most common extra-intestinal manifestations and complications of inflammatory bowel disease (IBD). This study aimed to identify crosstalk genes between IBD and osteoporosis (OP) and potential relationships between crosstalk and neutrophil extracellular traps (NETs)-related genes. Three common hub genes from different compared groups are actually the same, namely HDAC6, IL-8, and PPIF. ROC showed that the combined diagnostic value of HDAC6, IL-8, and PPIF was higher than each of the three key hub genes. Immune infiltration results showed that HDAC6 and IL-8 key genes negatively correlated with CD65 bright natural killer cells. USF1 was the common upstream TFs between HDAC6 and PPIF, and MYC was the common upstream TFs between IL-8 and PPIF in RegNetwork. Taken together, this study shows a linked mechanism between IBD and OP via NETs and crosstalk genes. These findings may show light on better diagnosis and treatment of IBD complicated with OP.

## Introduction

Inflammatory bowel disease (IBD) is one of the chronic and reduplicative gastrointestinal diseases with a high prevalence^1^. There are more than 2.5 million IBD patients in Europe, and more and more IBD patients in Asia^2^. Both ulcerative colitis (UC) and Crohn’s disease (CD) belong to IBD. In the process of IBD, over half of the patients can also have extraintestinal manifestations^3^. In this regard, above 50% of IBD patients suffer from osteoporosis (OP), which is a systemic metabolic disease and has become a major concern with the subsequent risk of fractures^4^. Some studies have reported bone alterations in IBD^5,6^. And a systematic review and meta-analysis indicated that there was a negative correlation between UC and bone mineral density^7^. Moreover, nutrients such as vitamins A, K, C, and B12, folic acid, calcium, and many others played important roles in the prevention of OP in IBD^8^. Emerging pieces of evidence exhibit that IBD affects OP, and they are correlative to some degree. However, these reports have failed to clarify the inherent relationship between IBD and OP.

The causes of OP are complex, it may be involved in chronic systemic inflammation^9,10^. Inflammatory cytokines such as tumor necrosis factor-α (TNF-α) and interleukins (ILs) may affect bone remodeling including promoting osteoclastic activity and reducing osteoblastic activity by up-regulating the receptor activator of NF-kappa B ligand^11–13^. And the activated immune response by inflammatory cytokines can be an important risk for OP^14^. In this context, IBD with the duration of intestinal chronic inflammation, contributes to systemic inflammation and bone loss. Thus, it is believed that inflammation may be an essential mechanism for IBD complicated with OP. 

The chronic inflammatory response in IBD is related to intestinal mucosal injury and intestinal epithelial barrier dysfunction, which consequently activates the immune response and promotes the extensive recruitment of neutrophils^15^. Neutrophils can enhance the permeability of intestinal epithelial, destruct the tissue through proteolytic and oxidative damage, and release inflammatory factors^16^. It has been evidenced that the massive infiltration of neutrophils is related to systemic inflammatory indexes in IBD^17,18^. Neutrophils can release their lytic granules and extracellular neutrophil traps (NETs)^19^. NETs produced from activated neutrophils are web-like structures and consist of decondensed chromatin DNA, histones, and the contents of granules^15,20^. NETs energetically participate in the pathogenesis of inflammatory diseases such as IBD, including increased inflammatory mediators, impaired epithelial barrier function, increased extracellular matrix degradation, increased proteolytic activity, and impairment^15^. However, the effects of NETs on OP have not yet been found, and the connection between them should be explored. Consequently, the link between IBD and OP combined with NETs deserves further investigation. It can advance the comprehension of the pathophysiological mechanisms underlying the development of IBD complicated with OP for better diagnosis and treatment.

In this study, bioinformatics was used to solve the puzzle in the joint clinical research of IBD and OP. We predict the key genes in the relationship between IBD and OP and their related signal pathways and investigate the mechanisms of the link between the two diseases by hunting for the crosstalk genes between IBD and OP and connecting them with NETs-related genes using correlation analysis and PPI network. 

We believe that NETs are one of the mutual mechanisms of IBD and OP. There is a link between crosstalk and NET-related genes in IBD and OP, and they can affect each other through bridge genes. Three crosstalk-NETs genes are strongly related in both diseases, which interact with one another and affect their expressions to influence the progress of IBD and OP by some mechanisms.

## Materials and methods

### Data collection

IBD datasets (GSE169568)^21^ and OP datasets (GSE56814)^22^ were obtained from GEO (https://www.ncbi.nlm.nih.gov/geo/), which is a huge public functional genomics database.

### Differential expression analysis

Differentially expressed genes (DEGs) between the control group and sick group were identified using GEO2R (www.ncbi.nlm.nih.gov/geo/geo2r/) online analysis tool based on R packages (GEOquery and Limma). DEGs were defined as genes with p-value < 0.05 and |log2 fold change |> 1. Visualize DEGs were presented as volcano plots and heatmaps using the ggplot2 package. Common DEGs both up-regulated or down-regulated between GSE169568 (UC and CD, respectively) and GSE56814 were extracted using the online Venn diagram tool.

### Functional enrichment analysis

To identify DEGs of UC and OP, DEGs of CD and OP, crosstalk genes and genes of key clusters, Gene Ontology (GO) annotation and Kyoto Encyclopedia of Genes and Genomes (KEGG)^23–25^ pathway enrichment analyses were performed using p-value < 0.05, min(overlap) = 2 and min(enrichment) = 1.5 were as thresholds. The enriched GO and KEGG pathways were selected and visualized using bubble plots.

### Crosstalk genes analysis

After identifying DEGs of UC and OP, DEGs of CD and OP separately, the intersection of DEGs of UC and OP and the intersection of DEGs of CD and OP were obtained using R software, representing the main links between UC and OP, and between CD and OP, namely crosstalk genes.

### Correlation of crosstalk genes with NETs genes

There were 69 NETs genes according to the previous studies^26^. To explore the effect of NETs on regulating the association between IBD and OP, the expression profiles of NETs and crosstalk genes in the IBD and OP datasets were obtained and assessed by calculating Pearson coefficients. To complement the above correlation, KEGG was employed to identify and classify the common pathways between crosstalk and NETs genes using bubble plots. 

### PPI network and community discovery analysis

Based on the crosstalk genes with NETs genes, GeneMania (https://GeneMANIA.org) database was used for protein–protein interaction (PPI), p-value < 0.05 was considered significant.

### Machine learning for recognition of IBD-related hub genes

LASSO is a regression method for selecting a variable to improve the predictive accuracy and is also a regression technique for variable selection and regularization to improve the predictive accuracy and comprehensibility of a statistical model^27^. The “glmnet” R package was used to perform LASSO regression analysis using a tenfold cross-validation^28^. The binomial deviance curve was plotted versus log (lambda) to ensure the minimum lambda. When the lowest partial likelihood deviance was found, the optimal lambda and number of genes were obtained. The intersection genes of LASSO were considered candidate hub genes in AVC diagnosis.

### Immune infiltration analysis

To further exhibit the immune cell sight, the analytical platform CIBERSORT (https://cibersort.stanford.edu/) was applied to decipher immune cell infiltration profiles of discover. Pearson correlation analysis was adopted to recognize the link between different immune cell phenotypes and hub genes.

### Upstream regulators

To investigate upstream transcriptional regulatory transcription factors (TFs) and post-transcriptional regulatory micro-RNAs (miRNAs) of hubs, TFs-hubs and miRNAs-hubs interaction network analysis were performed respectively, using the NetworkAnalyst tool. The TFs-hubs and miRNAs-hubs interaction networks were established by the RegNetwork repositor databases. Cytoscape software was used to improve the quality of networks^29^. 

### Statistical analysis

The ROC curve and the calculation of AUC, as well as 95% CI, were established using SPSS Version 19.0 (IBM Corporation, Armonk, NY, USA). Student’s sample t-test was used to distinguish the ratio of different immune cells between the control and sick groups via GraphPad Prism (GraphPad Software, San Diego, CA, USA). p-Value < 0.05 was considered statistically significant.

## Results

### Identification of DEGs from OP, UC, and CD

**Figure 1:**
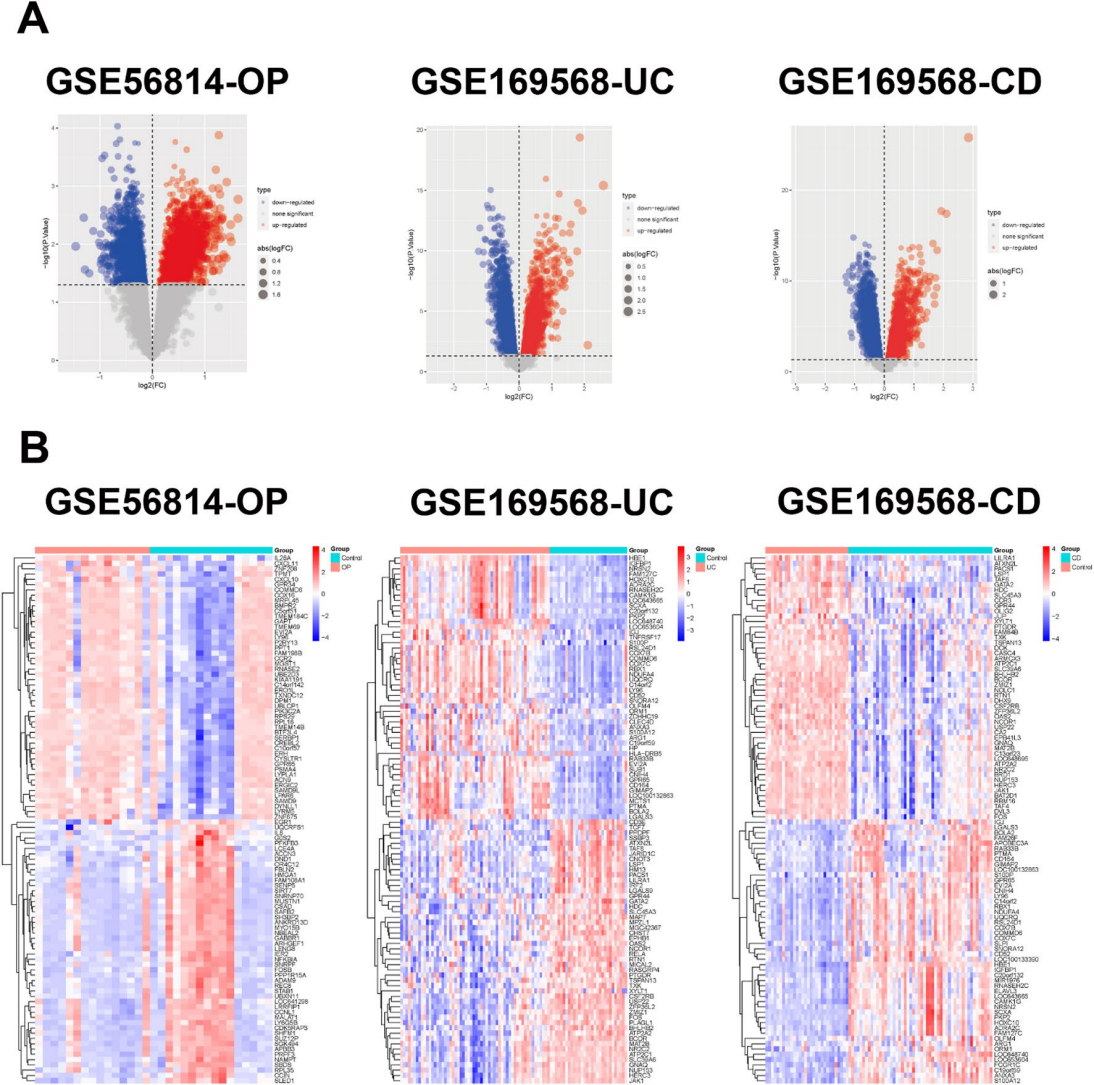
Volcano plot and heatmap for the DEGs identified from the datasets. (**A**) Red and blue plot dots represent DEGs with upregulated and downregulated gene expression, respectively. (**B**) Each row shows the DEGs, and each column represents the samples or controls. The red and blue represent DEGs with upregulated and downregulated gene expression, respectively. DEGs, differentially expressed genes. UC, ulcerative colitis. CD, Crohn’s disease. OP, osteoporosis.

The OP dataset (GSE56814) contained 16 control samples and 15 OP samples, and the IBD dataset (GSE169568) contained 30 control samples, 58 UC samples, and 52 CD samples. The samples were quantile-normalized using the preprocessCore R package (Supplementary Fig. 1). DEGs between control samples and OP in GSE56814, DEGs between control samples and UC in GSE169568, and DEGs between control samples and CD in GSE169568 were respectively analyzed using the Limma method and presented as volcano plots and heatmaps (Fig. 1).

### Identification and enrichment analysis of shared genes

**Figure 2:**
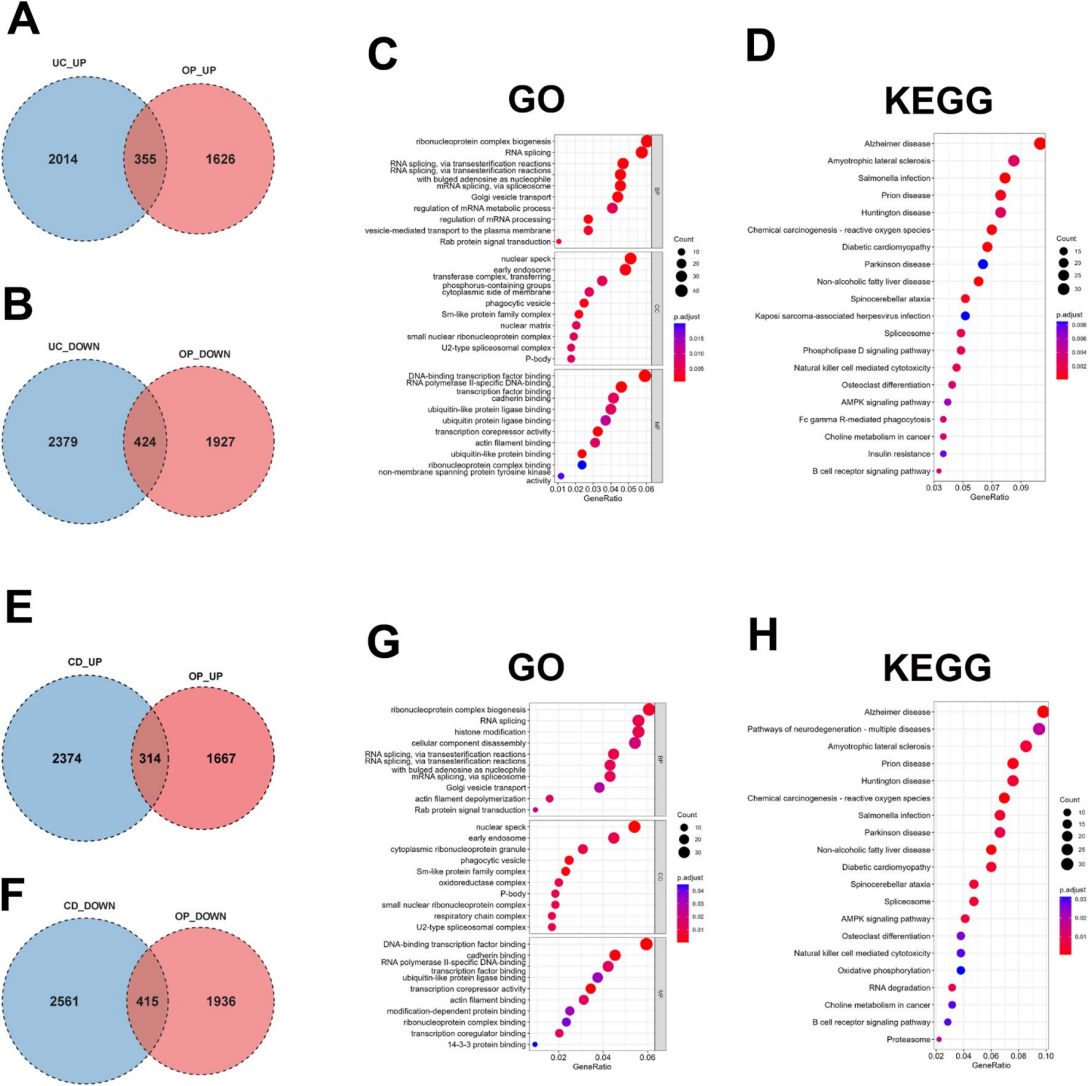
The shared genes in IBD and OP. (**A-B**) Venn diagram of the junction of DEGs in UC and OP. (**C-D**) GO and KEGG pathway enrichment analyses of crosstalk genes in UC and OP. (**E–F**) Venn diagram of the junction of DEGs in CD and OP. (**G-H**) GO and KEGG pathway enrichment analyses of shared genes in CD and OP. DEGs, differentially expressed genes. UC, ulcerative colitis. CD, Crohn’s disease. OP, osteoporosis. IBD, inflammatory bowel disease.

The junction of DEGs in UC and OP exhibited 779 shared genes, 355 of which were up-regulated and 424 of which were down-regulated in both UC and OP (Fig. 2A and B). To decode the biological functions of the above common DEGs, GO and KEGG pathway enrichment analyses were implemented on 779 crosstalk genes between UC and OP using the ClusterProfiler R package. GO analysis deciphered that common DEGs in the biological process (BP) mainly included “mRNA splicing, via transesterification reactions”, “mRNA splicing, via spliceosome”, “regulation of mRNA processing”, and others. As to cellular component (CC), common DEGs were chiefly enriched in “nuclear speck”, “early endosome”, “phagocytic vesicle”, and others. Molecular function (MF) analysis elucidated that “DNA-binding transcription factor binding”, “RNA polymerase II-specific DNA-binding” and “ubiquitin-like protein ligase binding” were the most obvious items in common DEGs (Fig. 2C). KEGG analysis showed that common DEGs were mainly focused on the “salmonella infection” and “prion disease” (Fig. 2D).

The joining of DEGs in CD and OP exhibited 729 crosstalk genes, 314 of which were up-regulated and 415 of which were down-regulated in both CD and OP (Fig. 2E and F). BP in GO analysis of Common DEGs showed that it mainly included “histone modification”, “mRNA splicing, via transesterification reactions”, “mRNA splicing, via spliceosome”, and others. CC analysis also exhibited that “nuclear speck”, “early endosome”, and “phagocytic vesicle” were the most significant selection, the same as common DEGs in UC and OP. With regard to MF, common DEGs were primarily enriched in “DNA-binding transcription factor binding”, “cadherin binding”, “RNA polymerase II-specific DNA-binding”, and others (Fig. 2G). KEGG analysis exhibited that common DEGs were mostly enriched in “amyotrophic lateral sclerosis” and “prion disease” (Fig. 2H).

### Identification of shared genes with NET-related genes

**Figure 3:**
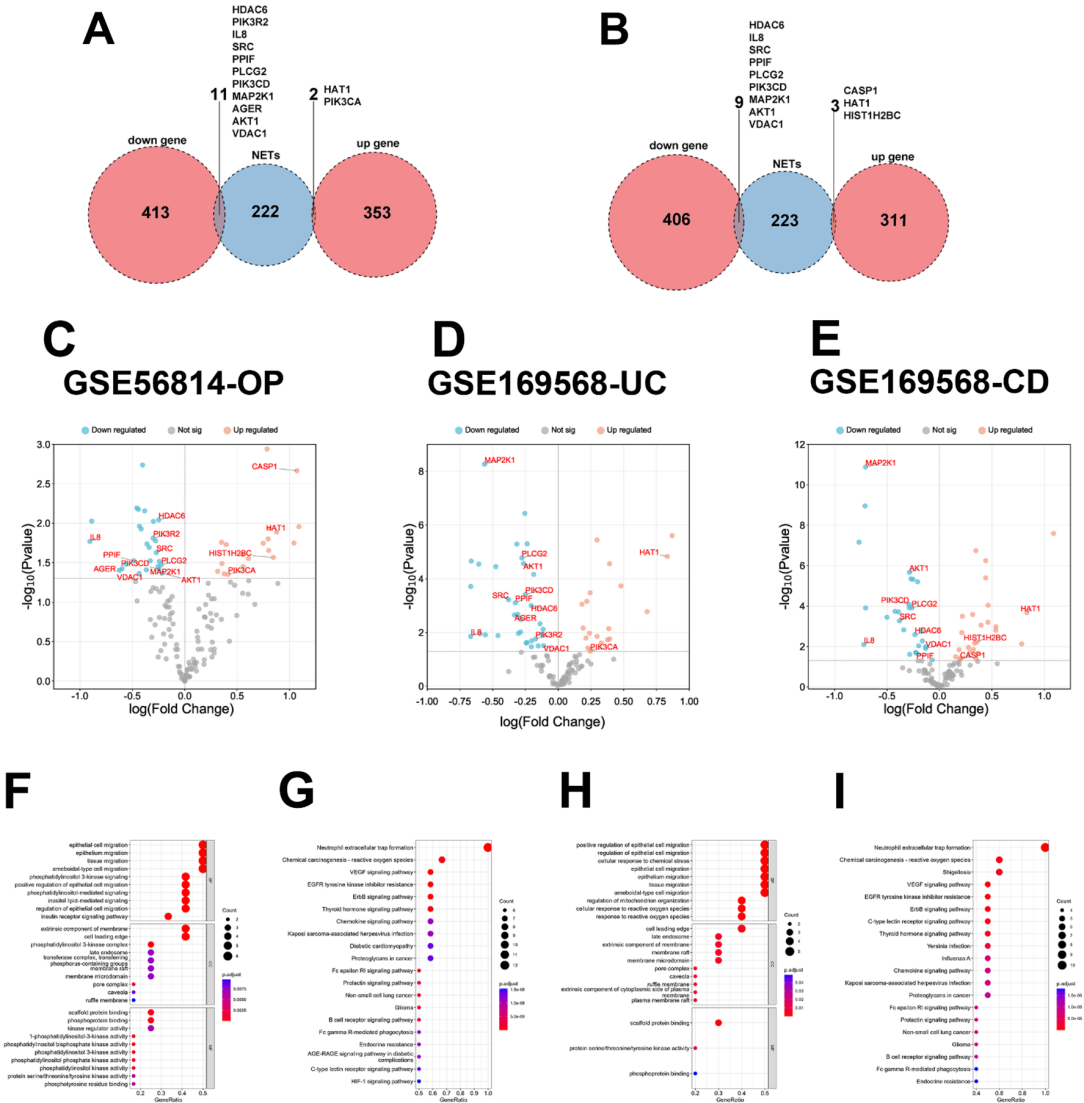
The common NET-related genes in IBD and OP. (**A**) Venn diagram of the interaction of common DEGs in UC and OP and NET-related genes. (**B**) Venn diagram of the interaction of common DEGs in CD and OP and NET-related genes. (**C-E**) Volcano plots for NET-related genes and the location of differentiated NET-related genes in the OP dataset (GSE56814), the UC dataset (GSE169568), and the CD dataset (GSE169568), respectively. (**F-G**) GO and KEGG pathway enrichment analyses of the differentiated NET-related genes in UC and OP. (**H-I**) GO and KEGG pathway enrichment analyses of the differentiated NET-related genes in CD and OP. DEGs, differentially expressed genes. NET, extracellular neutrophil trap. UC, ulcerative colitis. CD, Crohn’s disease. OP, osteoporosis. IBD, inflammatory bowel disease.

The interaction of common DEGs in UC and OP and NET-related genes exhibited 13 shared differentiated genes, 2 of which were up-regulated, namely HAT1 and PIK3CA, and 11 of which were down-regulated, namely HDAC6, PIK3R2, IL-8, SRC, PPIF, PLCG2, PIK3CD, MAP2K1, AGER, AKT1 and VDAC1 (Fig. 3A). Similarly, the junction of common DEGs in CD and OP and NET-related genes exhibited 12 shared differentiated genes, three of which were up-regulated, namely CASP1, HAT1, and HIST1H2BC, and 9 of which were down-regulated, namely HDAC6, IL-8, SRC, PPIF, PLCG2, PIK3CD, MAP2K1, AKT1 and VDAC1 (Fig. 3B). It was not hard to find that HAT1 was the only same up-regulated gene and HDAC6, IL-8, SRC, PPIF, PLCG2, PIK3CD, MAP2K1, AKT1, and VDAC1 were the common down-regulated genes whether in UC and OP or in CD and OP. Volcano plots (Fig. 3C–E) showed NET-related genes and the location of differentiated NET-related genes in the OP dataset (GSE56814), the UC dataset (GSE169568), and the CD dataset (GSE169568). GO and KEEG analyses were performed to decode 13 differentiated NET-related genes in UC and OP (Fig. 3F and G), and 12 differentiated NET-related genes in CD and OP (Fig. 3H and I).

**Figure 4:**
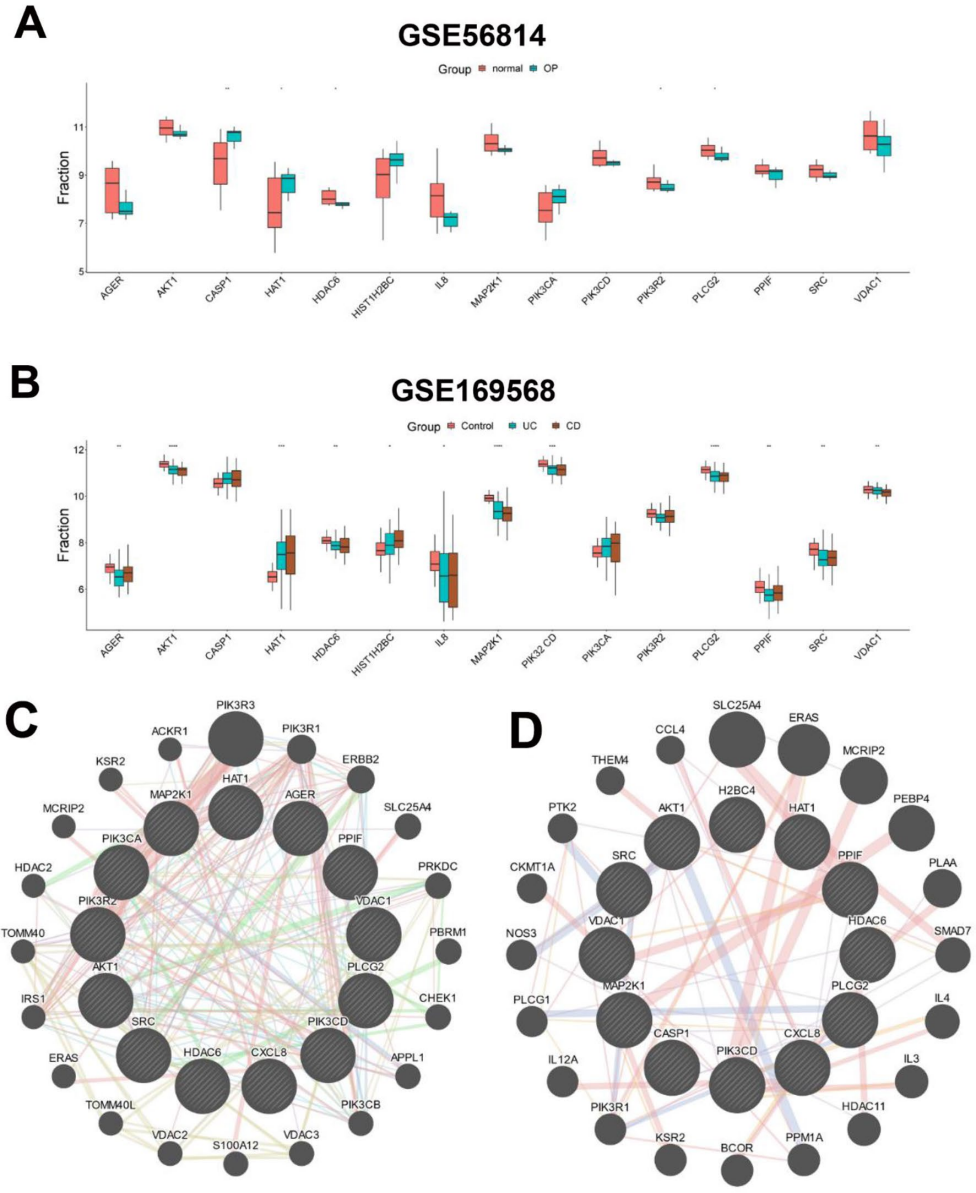
The differentiated NET-related genes. (**A**) Comparison of differentiated NET-related genes between the two groups (normal and OP) in GSE56814. (**B**) Comparison of differentiated NET-related genes among the three groups (control, UC, and OP) in GSE169568. *p-value < 0.05, **p-value < 0.01, ***p-value < 0.001, ****p-value < 0.0001. (**C**) The PPI network of 13 differentiated NET-related genes in UC and OP. (**D**) The PPI network of 12 differentiated NET-related genes in CD and OP. NET, extracellular neutrophil trap. UC, ulcerative colitis. CD, Crohn’s disease. OP, osteoporosis. PPI, protein–protein interaction.

All differentiated NET-related genes were also verified in two datasets, GSE56814 and GSE169568, most of which were obviously upregulated or downregulated in OP, UC, and CD (Fig. 4A and B). In order to further investigate the links between proteins coded by the shared differentiated NET-related genes of UC and OP or CD and OP, the protein–protein interaction (PPI) network of 13 differentiated NET-related genes in UC and OP or 12 differentiated NET-related genes in CD and OP, was analyzed with geneMANIA (www.genemania.org) and visualized with Cytoscape. It revealed that 20 genes could interact with 13 differentiated NET-related genes in UC and OP, 20 genes could interact with 12 differentiated NET-related genes in CD and OP, and 4 of 20 genes were the same, namely PIK3R1, SLC25A4, ERAS, and MCRIP2 (Fig. 4C and D).

### Identification of shared diagnostic genes of IBD and OP

**Figure 5:**
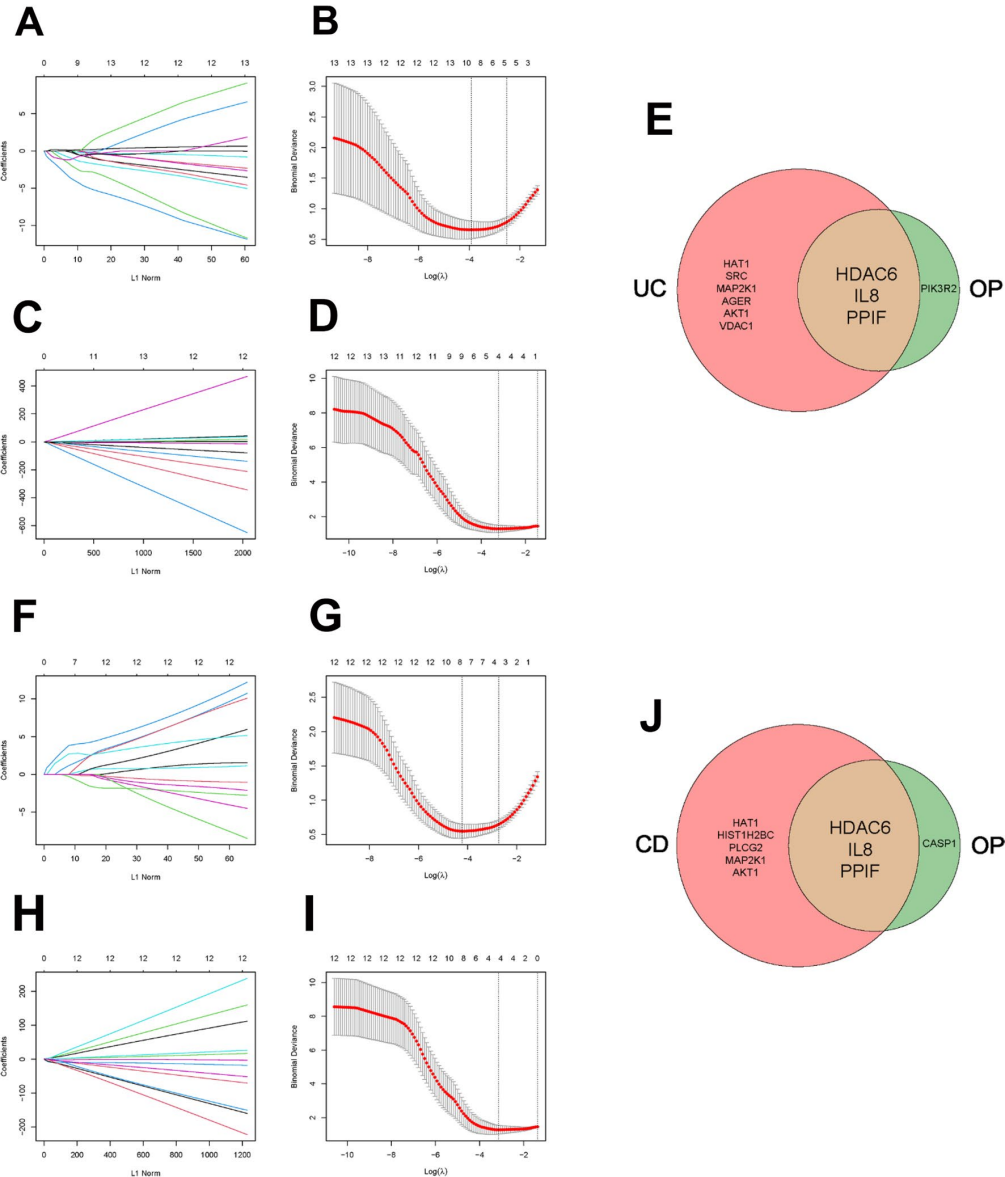
The diagnostic genes of IBD and OP. (**A, B**) The differentiated NET-related genes of UC in the Lasso model in GSE169568. (**C, D**) The differentiated NET-related genes of OP in the Lasso model in GSE56814. (**E**) Venn diagram showing the optimal diagnostic genes of UC and OP. (**F, G**) The differentiated NET-related genes of CD in the Lasso model in GSE169568. (**H, I**) The differentiated NET-related genes of OP in the Lasso model in GSE56814. (**J**) Venn diagram showing the optimal diagnostic genes of CD and OP. NET, extracellular neutrophil trap. UC, ulcerative colitis. CD, Crohn’s disease. OP, osteoporosis. IBD, inflammatory bowel disease.

In order to further narrow the gene range and discover shared diagnostic genes of IBD with OP, a LASSO regression algorithm was utilized to further find the hub genes. In GSE169568, LASSO analysis identified 9 hub genes out of 13 differentiated NET-related genes (Fig. 5A and B). Then, LASSO identified 4 hub genes out of 13 differentiated NET-related genes in GSE56814 (Fig. 5C and D). At last, three hub genes (HDAC6, IL-8, and PPIF) were found to be the potential shared diagnostic genes (Fig. 5E). In line with this, 8 hub genes in GSE169568 (Fig. 5F and G) and 4 hub genes in GSE56814 (Fig. 5H and I) were identified respectively by LASSO regression, then, the same three hub genes (HDAC6, IL-8, and PPIF) were found to be the optimal selection (Fig. 5J). Taken together, HDAC6, IL-8, and PPIF were regarded as hub genes and promising shared diagnostic genes of IBD and OP.

### Assessment of diagnostic efficiency

**Figure 6:**
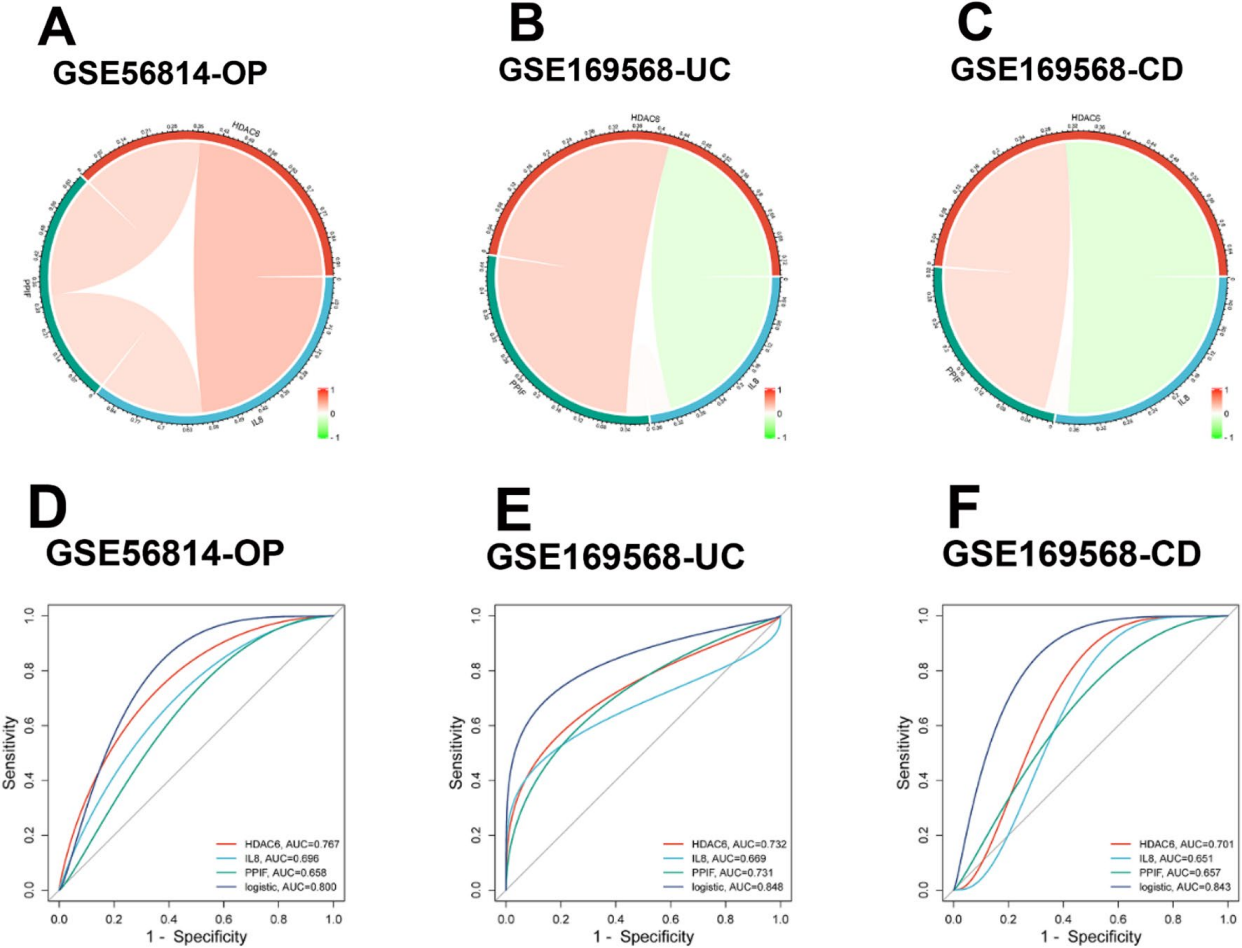
The hub genes of IBD and OP. (**A–C**) The correlation analysis of three hub genes in GSE169568 (UC and CD) and GSE56814 (OP). (**D–F**) ROC curves of three hub genes in diagnosing OP (**D**), UC (**E**) and CD (**F**). ROC, receiver operating characteristic. UC, ulcerative colitis. CD, Crohn’s disease. OP, osteoporosis. IBD, inflammatory bowel disease.

The correlation analysis of three hub genes in GSE169568 and GSE56814 was performed using the Circlize package in R. It revealed that HDAC6 had an obvious positive correlation in both GSE169568 and GSE56814 while PPIF had a significant negative correlation in both datasets (Fig. 6A–C). Moreover, ROC curves were constructed to assess whether HDAC6, IL-8, and PPIF had the diagnostic efficiency in the two testing datasets GSE169568 (UC and CD, IBD) and GSE56814 (OP). The AUC values of all three hub genes in both two datasets were greater than 0.6, the AUC values of HDAC6 in both two datasets were all greater than 0.7, and the AUC values of the logistic regression model consisting of HDAC6, IL-8, and PPIF in both two datasets were at least 0.8 (Fig. 6D–F). HDAC6 and the logistic regression model presented a high diagnostic efficiency for IBD with OP, and the latter had the highest diagnostic efficiency.

### Analysis of immune cell infiltration

**Figure 7:**
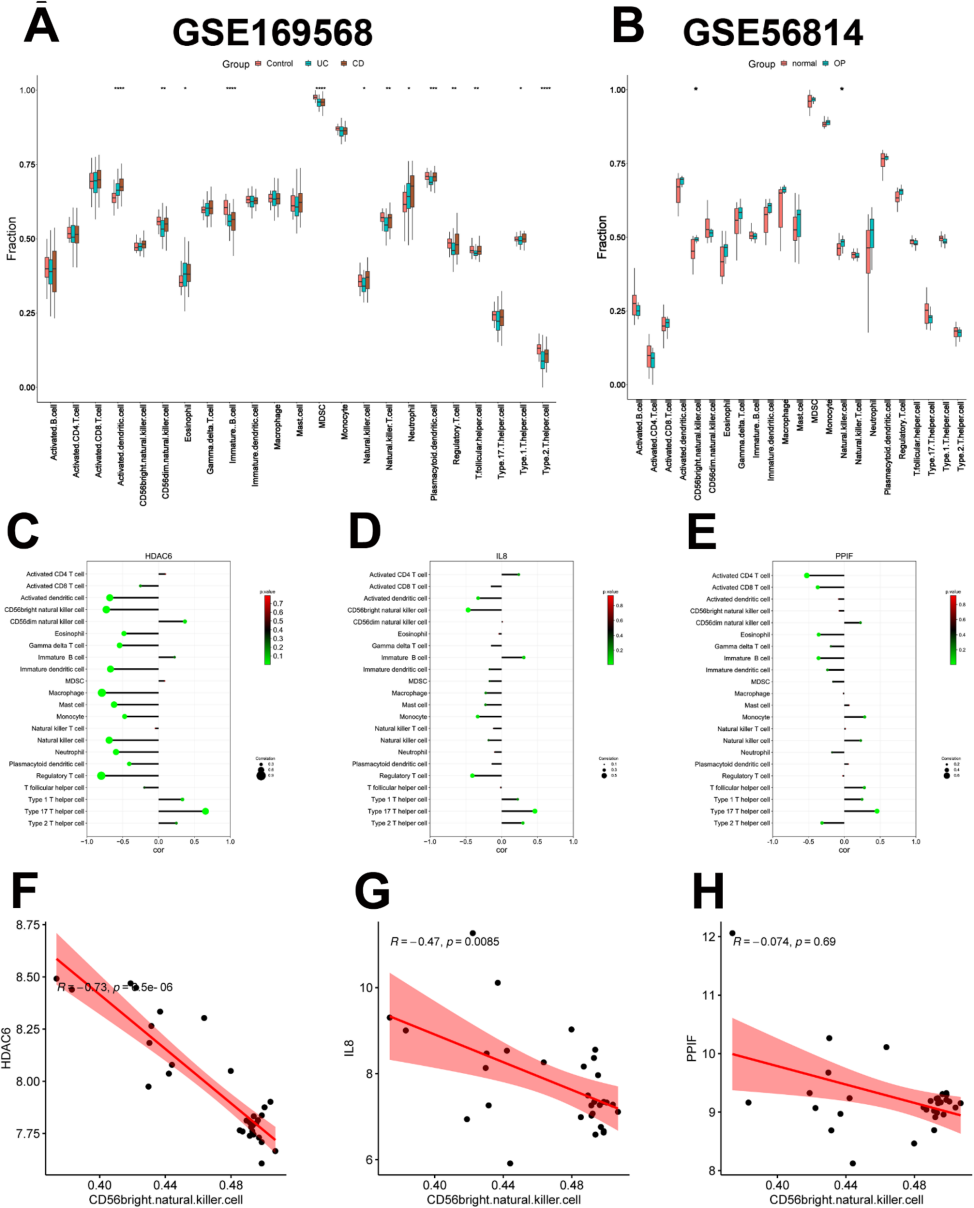
Immune cell infiltration. (**A, B**) The distribution of 23 immune cells in GSE159568 and GSE56814, respectively. (**C–E**) The correlation between HDAC6, IL-8, and PPIF with 22 immune cells in GSE56814 (OP), respectively. (**F–H**). The correlation between HDAC6, IL-8, and PPIF with CD56 bright natural killer cells in GSE56814 (OP), respectively. OP, osteoporosis.

Due to our investigation of the effects of NETs on IBD with OP, immune regulation may be selected as the potential IBD with OP diagnostic biomarker, and infiltration analysis was used to clarify the immune regulation of IBD with OP better. Immune cell infiltration differences of UC and CD in GSE169568, and OP in GSE56814 were displayed compared with control samples. Among 23 kinds of immune cells, the ratio of 13 kinds of immune cells was obviously different in GSE169568 (IBD), while the ratio of 2 kinds was markedly diverse in GSE56814 (OP), namely CD56 bright natural killer cell and natural killer cell (Fig. 7A and B).

In OP samples of GSE56814, the correlation between HDAC6, IL-8, and PPIF with 22 immune cells was respectively displayed. It demonstrated that HDAC6 and IL-8 were negatively correlated with CD56 bright natural killer cells and regulatory T cells, and HDAC6, IL-8, and PPIF were all positively linked with type 17 T helper cells (Fig. 7C–E). Furthermore, in OP samples of GSE56814, the correlation between HDAC6, IL-8, and PPIF with CD56 bright natural killer cells was respectively calculated. Consistent with the above results, HDAC6 (R = −0.73, p < 0.001) and IL-8 (R = −0.47, p < 0.01) were negatively correlated with CD56 bright natural killer cells while the link between PPIF with CD56 bright natural killer cells was not significant (R = −0.074, p = 0.69) (Fig. 7F–H). Taken together, various types of immune cells were distinguishingly infiltrated in IBD and OP patients, which may be regarded as promising therapeutic targets for IBD with OP.

### Identification of upstream regulators

**Figure 8:**
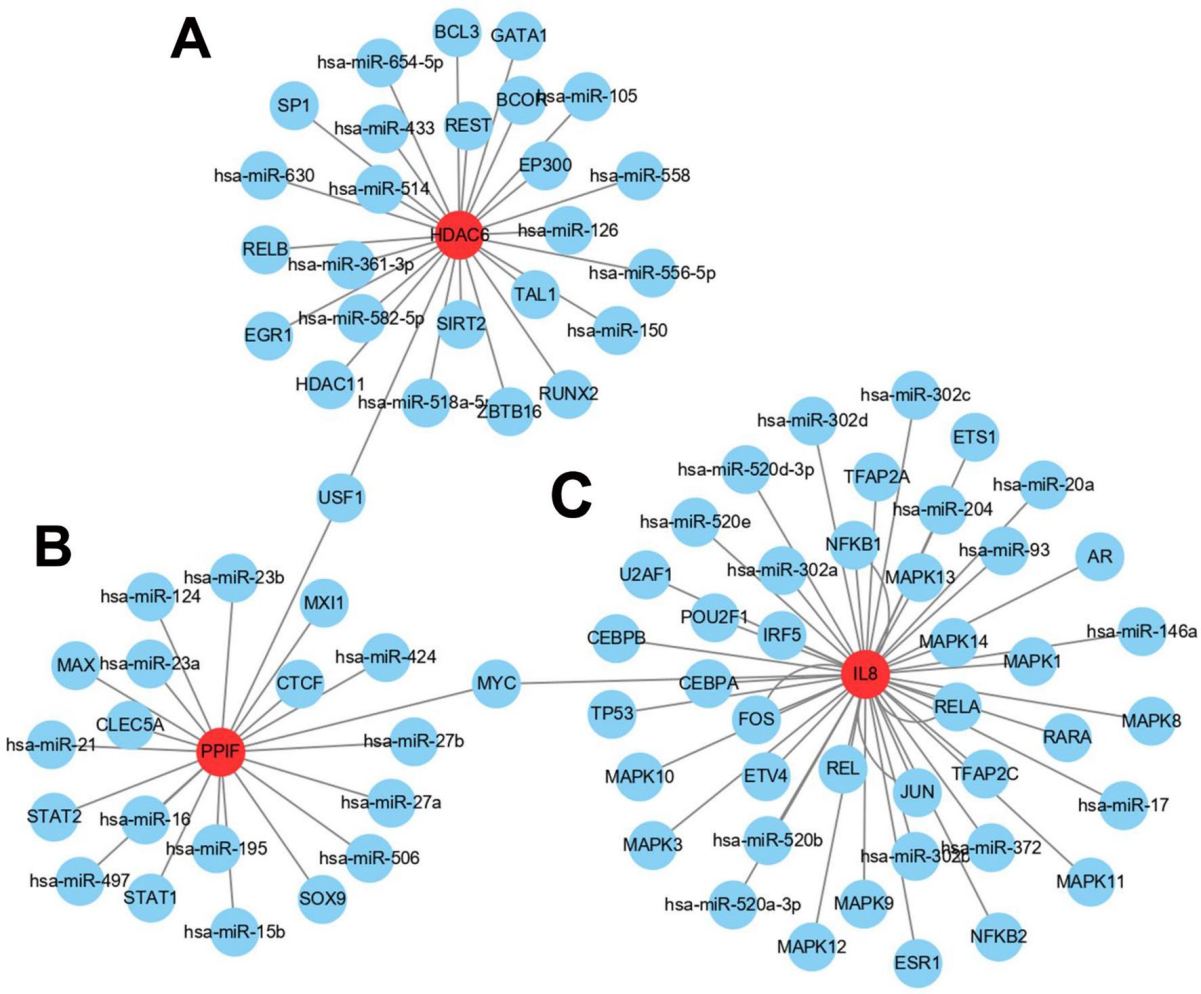
Upstream regulators. (**A–C**) Upstream TFs and post-transcriptional regulatory miRNAs of HDAC6, PPIF, and IL-8, respectively. TFs, transcription factors.

For the purpose of predicting upstream transcriptional regulatory TFs and post-transcriptional regulatory miRNAs of the three hubs, NetworkAnalyst and Cytoscape were used to construct the network and be presented visually. A total of 26 upstream TFs and miRNAs of HDAC6 were estimated. The upstream numbers of PPIF and IL-8 were 20 and 42, separately. In addition, it revealed that USF1 was the common upstream TFs between HDAC6 and PPIF, and MYC was the common upstream TFs between IL-8 and PPIF in RegNetwork (Fig. 8). USF1 and MYC may be the important upstream TFs to the three hub genes.

## Discussion

This study explored the link between IBD, OP, and NETs via bioinformatics, community detection, and machine learning. Three hub genes (HDAC6, IL-8, and PPIF) belong to common differentiated NET-related genes and were found as the main diagnostic genes to elucidate the relationship between IBD and OP, and USF1 and MYC may be the important upstream TFs to the three hub genes. 

Since both IBD and OP possess a link with inflammation, IBD and OP are potentially related. Inflammatory diseases are usually linked with OP. Inflammatory factors such as both pro-inflammatory mediators and chemokines act on the different kinds of bone cells^10,12,14,30^. More and more emerging evidence suggests that there must be a certain internal link between IBD and OP^3,5,7^. In this regard, the immune response and histone modification have recently been the research hotspots, in the mutual etiological factors of IBD and OP^31,32^. The immune system is inseparably associated with bone balance. The term “immunoporosis” has been coined to highlight the effects of the immune response on OP pathogenesis^30^. The abnormalities of the immune response have been confirmed to be imputed to improper histone modifications. Histone modifications including phosphorylation, ubiquitination, sumoylation, methylation, and acetylation belong to changing transcriptional activity^33^. For the current study, shared genes of CD and OP mainly take part in BP such as “histone modification”, identifying the relationship between IBD and OP. It’s worth noting that shared genes play important roles in histone modifications drawn into immune response, in line with previous research and contributing to mutual immune-inflammatory mechanisms in both illnesses.

A remarkable inflammatory course actuated by neutrophils is regulated through their formed NETs. NETs are involved in numerous diseases, such as cancer, diabetes, rheumatoid arthritis, atherosclerosis, vasculitis, thrombosis, systemic lupus erythematosus, wound trauma and healing, and IBD^15,34,35^. NETs can provoke the evolution of autoimmunity and other dysfunction^34^. The formation of DNA-based NETs can be induced by the classical activator phorbol 12-myristate 13-acetate (PMA). Raloxifene, an estrogen receptor regulator, anti-inflammatory and anti-OP compound, can prevent NETs formation induced by PMA and neutrophil cell death^36^. However, the effects of NETs on OP remain obscure. NETs have been believed in playing a pivotal role in OP in one study concerning the relationship between periodontitis and OP based on bioinformatics and machine learning^37^. Thus, the underlying role of NETs in IBD and OP is also worth investigating. 

Intriguingly, in this study, HAT1 was the up-regulated shared NET-related gene and HDAC6, IL-8, SRC, PPIF, PLCG2, PIK3CD, MAP2K1, AKT1, and VDAC1 were the down-regulated shared NET-related genes in IBD and OP. Histone acetyltransferase 1 (HAT1) is one of the Gcn5-related N-acetyltransferase family and a type B histone acetyltransferase, which is involved in cancer, viral infections, immunoinflammatory and vascular diseases^38^. SRC, the protein tyrosine kinase, is associated with cancer and OP^39^. Phospholipase C γ 2 (PLCG2) 2 is reportedly involved in the inflammatory response and Alzheimer’s disease^40^. PIK3CD encodes the PI3K catalytic subunit, p110δ, a lipid kinase associated with neurodevelopmental dysregulation, which is affected by proinflammatory cytokine signaling^41^. Voltage-dependent anion channel 1 (VDAC1), situated on the mitochondrial outer membrane, affects inflammatory responses and Ca^2+^ transportation^42^, and is also involved in Alzheimer’s disease^43^.

Furthermore, three hub genes (HDAC6, IL-8, and PPIF) out of the above shared NET-related genes were selected as diagnostic genes of IBD with OP. The AUC values of HDAC6 in both two datasets were greater than 0.7, which was higher than those of IL-8, and PPIF. And the integrated AUC values of the three genes were 0.8, which was the highest. As one of the eleven zinc-containing HDAC enzymes involved in immune responses and other processes, HDAC6 has been reported to play a pivotal pro-inflammatory role in IBD^44^. The inhibitor of HDAC6 could restrain B-cell infiltration in DSS-induced colitis in mice^45^. Another inhibitor of HDAC6 also exhibited protective effects on colitis in mice by preventing NLRP3 inflammasome activation^46^. With regard to OP, HDAC6 could inactivate the Runx2 promoter to prevent osteogenesis of bone marrow stromal cells in age-related bone loss of mice^47^, osteocalcin expression could be upregulated by joint inhibition of HDAC6 and glucocorticoid receptors during osteogenic differentiation of mesenchymal stromal cells^48^. In MC3T3‑E1 cells, an HDAC6 inhibitor reduced the dexamethasone‑induced osteogenesis restriction^49^. Taken together, the suppression of HDAC6 can protect from both IBD and OP, it may also be a solid target for early diagnosis and treatment of IBD with OP. 

Cyclophilin D (CypD), encoded by the nuclear gene PPIF, is a chaperone protein and is associated with protein folding^50^. It was evidenced that the downregulation of CypD regulated by BMP signaling was significant during the osteogenic differentiation and it was harmful to the phenotype and strength of bone that increased CypD expression in osteoblasts during aging^51^. It was also reported that the reduction in bone formation could be blocked in CypD knock-out mice^52^. For IBD, an enhancer including an IBD risk variant could control PPIF expression to coordinate mitochondrial membrane potential inside macrophages^53^.

Interleukin-8 (IL-8) characteristics have been investigated, which was found as a chemical inducer for granulocytes, mainly neutrophils in vitro. IL-8 can be produced and secreted in the cells with Toll-like receptors such as macrophages. IL-8 is thought to be a strong inducer of cell migration and proliferation in inflammation models^54^. With regard to IBD, neutrophils in the patients’ blood exhibited superfluous IL-8, which was associated with glucose-6-phosphatase catalytic subunit 3 deficiency^55^. IL-8 has also been involved in bone remodeling and OP, IL-8 levels were obviously elevated in post-menopausal women with OP and bone loss. Atorvastatin could low IL-8 levels and reduce bone loss in osteoporotic rats induced by glucocorticoid^54^. 

Furthermore, upstream transcriptional regulatory TFs and post-transcriptional regulatory miRNAs of the three hubs were investigated and analyzed. The numbers of upstream TFs and miRNAs of IL-8, HDAC6, and PPIF were 42, 26, and 20, respectively. And USF1 was the common upstream TFs between HDAC6 and PPIF, while MYC was the common upstream TFs between IL-8 and PPIF. The upstream stimulatory factor 1 (USF1), as a general transcription factor in mammalian cells, can bind to the E-box motif in the promoter regions of many genes^56^. USF1 was involved in hyperlipidemia and lipid levels^57^, osteoblast differentiation^58,59^, and inflammation^60^. Interestingly, the USF1-CHCHD4 axis can promote the development of lung adenocarcinoma by activating the MYC pathway^56^, indicating a potential link between USF1 and MYC. MYC is also a common transcription factor regulating cell metabolism, proliferation, and differentiation^61^. MYC is associated with IBD diagnosis^62^, osteoclast activation^63^, and osteoclastogenesis^64^. Taken together, USF1 and MYC may be the common upstream TFs of IL-8, HDAC6, and PPIF, and they may also interact with each other. The interaction between USF1 and MYC should be further investigated in IBD with OP.

### Limitation

In this study, there were some limitations. Firstly, we only selected two datasets, namely IBD (GSE169568) and OP (GSE56814). In GSE169568, there were only 30 control samples, 58 UC samples, and 52 CD samples, and in GSE56814, there were only 16 control samples and 15 OP samples. The samples in both datasets seemed not too much, and the diagnostic logistic regression model was so high thanks to the restricted sample capacity. A more large-scale study with a large sample capacity should be verified in the future. Secondly, the links between the three hub genes and disordered immune cell infiltration still need to be investigated, even though the three hub genes were primarily focused on immune pathways, inflammatory diseases, and OP.

## Conclusion

This study shows a linked mechanism between IBD and OP via crosstalk and NETs. The pivotal hub genes HDAC6, IL-8, and PPIF were associated with NETs and TFs, influencing both IBD and OP. The disordered immune cell infiltration in IBD with OP was also exhibited for further investigation. These findings may show light on better diagnosis and treatment of IBD complicated with OP.

### Supplementary Information

Supplementary Information.

## Data Availability

In this study, two publicly available datasets were analyzed. This data can be found at GEO data repository (https://www.ncbi.nlm.nih.gov/geo/).
