# Supplementary material for: Identification of neutrophil extracellular traps and crosstalk genes linking inflammatory bowel disease and osteoporosis by integrated bioinformatics analysis and machine learning
Source: Sci Rep. 2023 Dec 27;13:23054. doi: 10.1038/s41598-023-50488-4 (PMC10754907; doi:10.1038/s41598-023-50488-4)

**Supplementary Figure 1.**

Quantile-normalized samples. (A) Untreated samples in GSE56814 and GSE169568. (B) Quantile-normalized samples in GSE56814 and GSE169568.


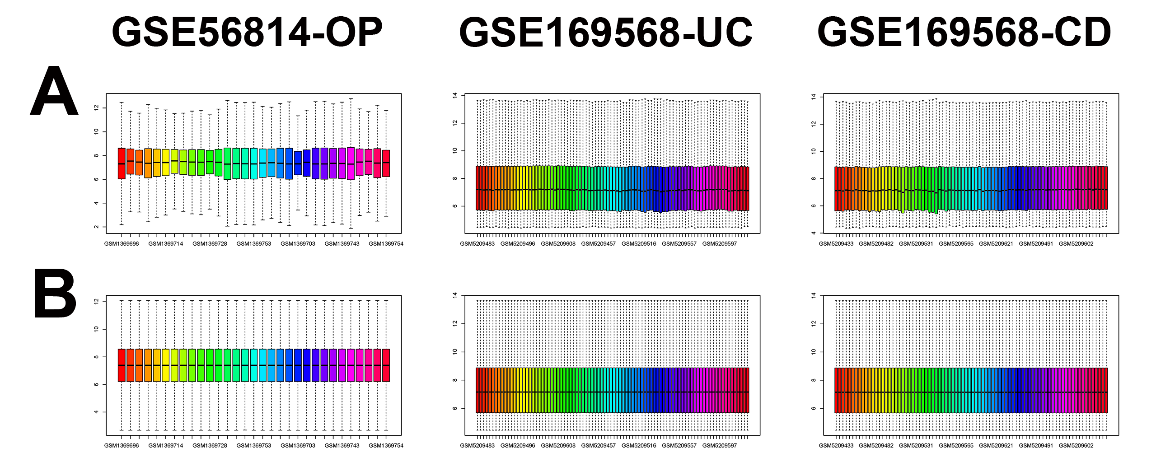

Supplement: Supplementary file 1 — Supplementary Information. [file 41598_2023_50488_MOESM1_ESM.docx]
